# Serum Starvation Induced Cell Cycle Synchronization Facilitates Human Somatic Cells Reprogramming

**DOI:** 10.1371/journal.pone.0028203

**Published:** 2012-04-18

**Authors:** Mengfei Chen, Jingjing Huang, Xuejiao Yang, Bingqian Liu, Weizhong Zhang, Li Huang, Fei Deng, Jian Ma, Yujing Bai, Rong Lu, Bing Huang, Qianying Gao, Yehong Zhuo, Jian Ge

**Affiliations:** 1 State Key Laboratory of Ophthalmology, Zhongshan Ophthalmic Center, Sun Yat-sen University, Guangzhou, Guangdong, China; 2 Department of Microbiology and Molecular Genetics, University of Pittsburgh School of Medicine, Pittsburgh, Pennsylvania, United States of America; Federal University of Rio de Janeiro, Brazil

## Abstract

Human induced pluripotent stem cells (iPSCs) provide a valuable model for regenerative medicine and human disease research. To date, however, the reprogramming efficiency of human adult cells is still low. Recent studies have revealed that cell cycle is a key parameter driving epigenetic reprogramming to pluripotency. As is well known, retroviruses such as the Moloney murine leukemia virus (MoMLV) require cell division to integrate into the host genome and replicate, whereas the target primary cells for reprogramming are a mixture of several cell types with different cell cycle rhythms. Whether cell cycle synchronization has potential effect on retrovirus induced reprogramming has not been detailed. In this study, utilizing transient serum starvation induced synchronization, we demonstrated that starvation generated a reversible cell cycle arrest and synchronously progressed through G2/M phase after release, substantially improving retroviral infection efficiency. Interestingly, synchronized human dermal fibroblasts (HDF) and adipose stem cells (ASC) exhibited more homogenous epithelial morphology than normal FBS control after infection, and the expression of epithelial markers such as E-cadherin and Epcam were strongly activated. Futhermore, synchronization treatment ultimately improved Nanog positive clones, achieved a 15–20 fold increase. These results suggested that cell cycle synchronization promotes the mesenchymal to epithelial transition (MET) and facilitates retrovirus mediated reprogramming. Our study, utilization of serum starvation rather than additional chemicals, provide a new insight into cell cycle regulation and induced reprogramming of human cells.

## Introduction

Ectopic expression of reprogramming factors can drive human somatic cells to return to embryonic stem cells (ESCs) like state [1], [2], this synthetic population are termed induced pluripotent stem cells (iPSCs). Human iPSCs display the features of self-renewal and the potential to differentiate into three germ layers, which holds great promise for regenerative medicine and human disease research [3].

To date, however, reprogramming of human adult cells is still challenging and inefficient. A number of studies have identified small molecules that can enhance reprogramming, such as the DNA methyltransferase inhibitor AZA [4], histone deacetylase inhibitor valproic acid [5], ALK5 inhibitor SB431542, MEK inhibitor PD0325901 [6], antioxidant vitamin C [7], etc. Other strategies include activation of the Wnt signaling pathway by Wnt3a [8] and inhibition of the p53/p21 pathway [9]. These findings suggested that multiple signaling pathways are involved in reprogramming.

Activation of endogenous pluripotency-related genes and epigenetic changes are significant markers of successful reprogramming [10]. To achieve these standards, consistent expression of the reprogramming factors is essential. In recent research, the MoMLV-based retrovirus vector has been a popular strategy for ectopic expression of reprogramming factors [1], [11]–[13]. However, the random viral integration results in genetic heterogeneity in the infected cell culture, which likely contributes to the low efficiency of reprogramming [14].

It is well known that the host cell cycle plays an essential role in retroviral infection. Retroviruses such as MoMLV require the disassembly of the nuclear envelope at mitosis to enter the nucleus and replicate [15]. Accurate study of host cell-retrovirus interaction has established that the integration of viral DNA occurs only after the infected cell has progressed through mitosis [16]. Notably, mitosis phase is much shorter than interphase, lasting only 20 mins in human fibroblasts [17]. Furthermore, the MoMLV vector derived retrovirus is not stable; the extracellular and intracellular half-life are 6–8 h and 5.5–7.5 h, respectively [18], [19]. However, the primary culture of HDF or ASC is a mixture of stem cells, progenitor cells and adult cells with their respective cell cycles [1], [20]. It is therefore not surprising that a large population of cells will escape from retroviral infection if the cell cycle does not arrive at mitosis.

Given that retroviral infection-mediated reprogramming involves host cell division, we speculate that a homogeneous and high efficiency infection may be achieved by cell cycle synchronization, which would probably be followed by the promotion of reprogramming. In this study, we used serum starvation induced cell cycle synchronization to accumulate cell population prior to G2/M, substantially improved retroviral infection efficiency. Furthermore, synchronization before reprogramming factors (OCT3/4, SOX2, C-MYC, and KLF4) transduction clearly promoted the MET, which in turn facilitated reprogramming. Our findings provide a new insight into cell cycle regulation and the reprogramming of human cells.

## Results

### 1. Serum starvation induced cell cycle synchronization

HDF and ASC are readily accessible cell sources and have been extensively used for reprogramming study [1], [21]. In present study, HDF and ASC were used to test the potential effect of cell cycle synchronization in reprogramming. To maintain a high proliferation rate, HDF and ASC were cultured in medium with 20% FBS (fetal bovine serum). Under this condition, the doubling times of HDF and ASC were 26±1.5 h and 28±2.2 h, respectively, which were similar to previous reports [22], [23].

**Figure 1:**
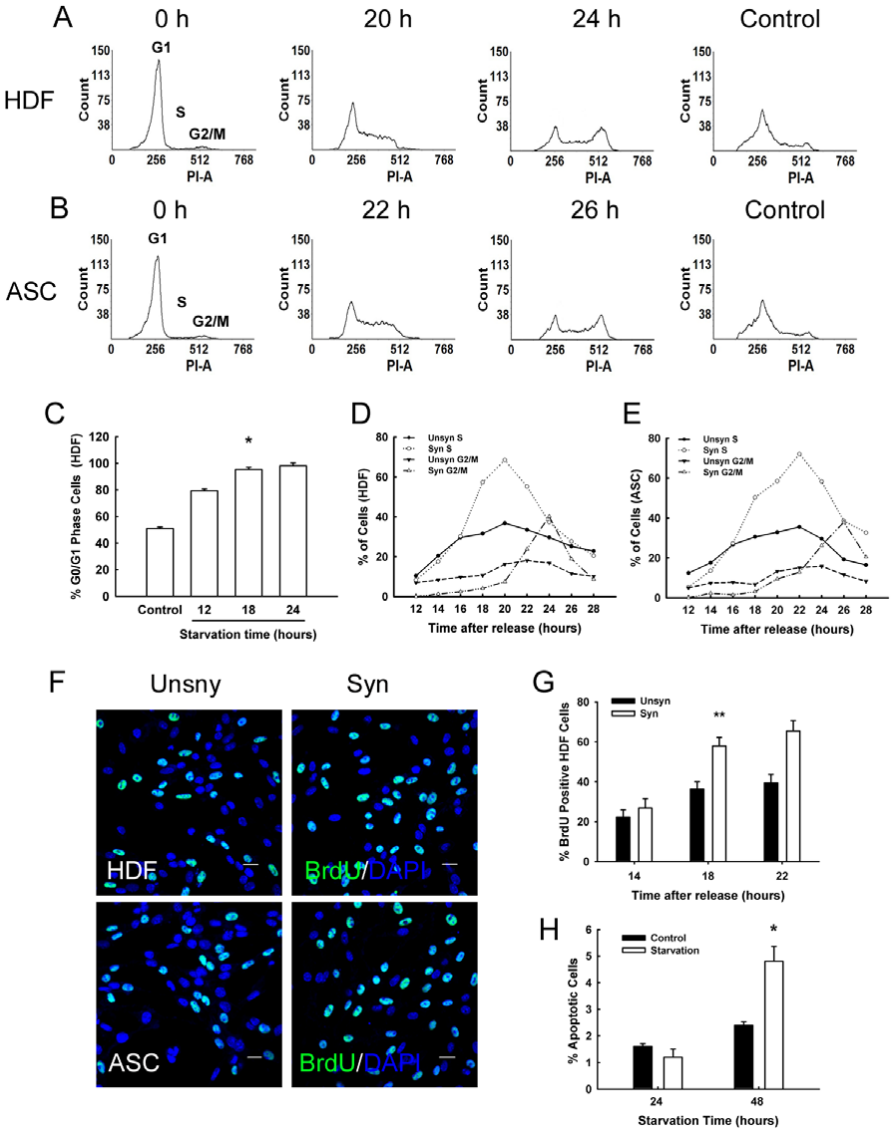
Serum starvation induced cell cycle synchronization. (A) and (B): Representative FACS data showed cell cycle arrested at G0/G1 phase (0 h) after serum starvation for 18 h. Re-feeding with FBS resulted in a clear accumulation of S and G2/M phase at the indicated time. (C): Modfit LT analysis revealed a time dependant increase in G0/G1 fraction upon serum deprivation. *P<0.01 *vs* control (n = 3). (D) and (E): A summary of cell cycle distribution after release from starvation. (F): Immunofluorescence revealed HDF and ASC increased BrdU incorporation after synchronization. Green fluorescence represents BrdU; the nuclei were labeled with DAPI (blue). Scale Bars = 20 µm. (G): Quantitative analysis indicated starvation and re-feed procedure increased BrdU positive cells in HDF. Counting was based on Brdu and DAPI staining. Positive cells were counted from a total number of at least 300 cells per well (n = 3) in randomly selected field. *P<0.01 *vs* unsynchronized control (n = 3). (H): Apoptosis assay of HDF after serum deprivation by Hoechst 33258 staining. *P<0.05 *vs* control (n = 3).

To define a feasible starvation period for synchronization, HDF and ASC were subjected to serum deprivation for 12, 18, or 24 h and thereafter resupplied with serum. The DNA content was analyzed by fluorescence activated cell sorter (FACS) at the end of each starvation period. DNA histogram revealed that over 95% of HDF were inhibited at G0/G1 phase after starvation for 18 h (Figure 1A, C). Analogous results were observed in ASC (Figure 1B, S1A). Starvation for 24 h did not achieve a more distinct inhibitory effect when compared with 18 h (Figure 1C).

After starvation, the cells were passaged and released into cell cycle by addition of serum [24]. Re-feeding with serum resulted in a clear enrichment of S (20 h) and G2/M (24 h) phase in HDF when compared with normal FBS supply control (Figure 1A, D). Similarly, accumulation of S and G2/M phase in ASC was detected at 22 h and 26 h, respectively (Figure 1B, E). These cell cycle profiles were in agreement with a previous study [25].

Using a BrdU incorporation assay, we next evaluated the cells that had progressed through DNA synthesis after release. Figure 1F showed representative images of BrdU staining. After release, synchronized HDF and ASC evidently increased the BrdU positive cells when compared with the controls. Quantitative analysis demonstrated that a significant increase in BrdU labeled population was detected at 18 h in HDF and 20 h in ASC after release (Figure 1G, S1B), corresponding to our FACS data. Taken together, these data further support that transient serum starvation induced a reversible cell cycle blocking and generated accumulation of S and G2/M fraction.

Previous study have shown that cell line 3T3 was sensitive to serum deprivation and inclined toward apoptosis [26]. In human foreskin fibroblasts, however, starvation for 32 h did not induce detectable apoptosis [27]. Our data also revealed that there was no significant difference between control and 24 h starved HDF in apoptosis and cell morphology (Figure 1H, S1C, D). Notably, Hoechst33258 staining detected that 48 h starvation caused appreciable apoptosis (Figure S1D). We therefore chose 18 h starvation in the following experiments to avoid the potential drawbacks of extended serum deprivation.

### 2. Cell cycle synchronization improved infection efficiency

**Figure 2:**
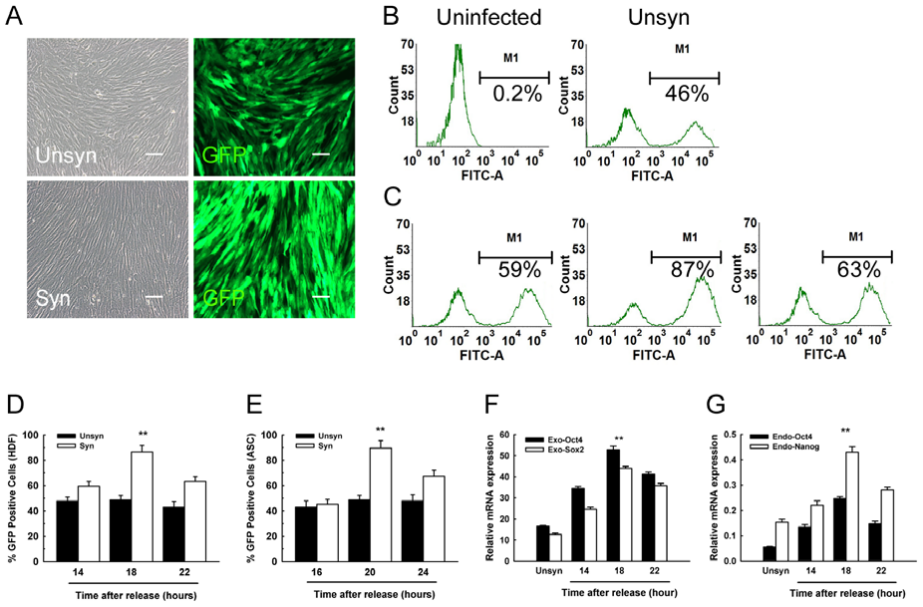
Cell cycle synchronization increased retrovirus mediated transduction. (A): Fluorescence microscope figures showed the improved infection of GFP in HDF after synchronization. To avoid misleading results from excessive virus infection, 0.1 ml concentrated GFP retrovirus was added to HDF in one well of a 12-well plate. The samples were analyzed at 72 h after infection. Scale Bars = 100 µm. (B): About 46% cells were GFP positive in unsynchronized HDF. A minimum of 30,000 events were acquired for each sample. (C): Representative FACS results showed that GFP positive HDF cells distinctly increased after synchronization and release for 14 h (left), 18 h (middle), and 22 h (right). (D) and (E): Statistical analysis of FACS data identified that the infection efficiency was enhanced about 1.9-fold in synchronized HDF and ASC. **P<0.01 *vs* unsynchronized control (n = 3). (F): Quantitative RT-PCR for transgene expression of Oct4 and Sox2 on day 5 after infection. **P<0.05 *vs* 22 h release (n = 3). (G): Quantitative RT-PCR for endogenous expression of Oct4 and Nanog on day 6 after OSKM (Oct4, Sox2, Klf4, and c-Myc) infection. **P<0.01 *vs* 22 h release (n = 3).

In view of the above findings, we speculated that retrovirus infected synchronized cells prior to G2/M peak could facilitate the transduction rate. To this end, we took advantage of a pMXs-GFP vector to generate GFP retrovirus and assess infection efficiency. HDF were serum deprived for 18 h and restimulated to enter cell cycle by addition of 20% serum [28] for 14–22 h before infection. Fluorescence images shown in Figure 2A revealed an evident increase of GFP positive cells in synchronized HDF after infection. Quantitative analysis by FACS demonstrated that about 46% cells were GFP positive in the unsynchronized control (Figure 2B). Notably, up to 87% GFP positive cells were achieved after infection at 18 h upon release (about a 1.9-fold increase, Figure 2 C, D). However, shortening (14 h) or prolonging (22 h) the release term resulted in infection decrease. These data are in agreement with our FACS results (Figure 1 D), as the cells need more time to enter (14 h) or part of cells have gone through G2/M phase (22 h). This improvement in infection rate was also observed in synchronized ASC (Figure 2E), about 89.6% ASC were GFP positive when infected at 20 h after release.

We next tested whether a synchronized population could elevate the expression of reprogramming factors after OCT3/4, SOX2, C-MYC, and KLF4 infection. Quantitative RT-PCR was performed to directly detect the expression of exogenous reprogramming factors on day 5 after infection. As expected, our results demonstrated that the expression of exogenous Oct4 and Sox2 was markedly enhanced by synchronization (Figure 2F). Given the fact that sustained transgenic expression is essential to activate pluripotency markers, we then measured the endogenous pluripotency genes on day 6 after infection. Quantitative RT-PCR results verified that expression of endogenous Oct4 and Nanog was detectable on day 6 in all retrovirus infected HDF. Notably, all the synchronized target cells exhibited a higher level of mRNA expression when compared with normal FBS controls. Furthermore, about a 5-fold increase in Oct4 expression and a 3-fold in Nanog expression were detected when infection started at 18 h after release (Figure 2G). These results suggested that transient serum starvation improved retroviral mediated gene transduction.

### 3. Synchronization procedure generated homogeneous MET and promoted reprogramming

**Figure 3:**
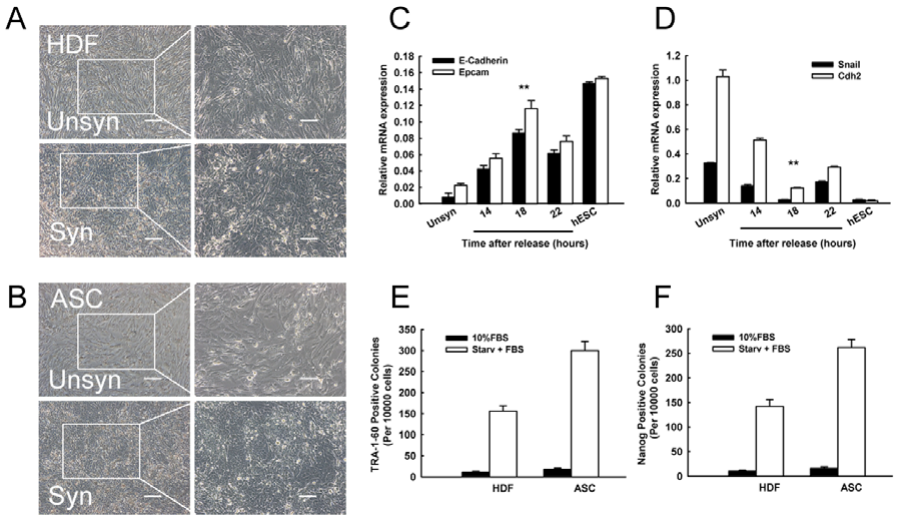
Synchronization procedure promoted MET and reprogramming. (A) and (B): Microscope images of HDF and ASC on day 6 after reprogramming factors infection. Higher magnification images of the boxed regions in (A) and (B) are shown in right. Scale Bars = 200 µm (left), 100 µm (right). (C): Quantitative RT-PCR revealed the up-regulation of epithelial markers in infected HDF. **P<0.05 *vs* 22 h release (n = 3). (D): The expression of mesenchymal associated genes was suppressed. *P<0.01 *vs* FBS control, **P<0.05 *vs* 22 h release (n = 3). (E) and (F): Synchronization increased TRA-1–60 and Nanog positive clones both in HDF and ASC. Counting was based on TRA-1–60 or Nanog plus DAPI staining; the data was collected from 3 independent experiments.

Recent studies have revealed that somatic cells have to undergo a reverse process from mesenchymal to epithelial transition (MET) during the early stages of reprogramming [29]. To investigate whether synchronization facilitates MET during human somatic cell reprogramming, we firstly captured images of the cell on day 6 after infection. Interestingly, a homogeneous epithelial morphology shift emerged in the synchronized HDF and ASC (Figure 3A, B). This conversion was distinguishable from the unsynchronized control, in which only a portion of the cells displayed epithelial-like morphology. Gene expression analysis confirmed that the reprogrammed cells in all groups showed activated expression of epithelial-related markers and downregulated mesenchymal key genes. Consistent with these morphological changes, serum starvation induced synchronization further improved the expression of epithelial markers E-cadherin (10-fold increase in 18 h release) and Epcam (6-fold increase in 18 h release) when contrasted with normal FBS controls (Figure 3C). At the same time, an apparent inhibition of Snail and Cdh2 expression was observed after synchronization (Figure 3D), consistent with our observations of the infection efficiency test. We therefore prefer 18 h release after starvation for reprogramming study.

We next asked whether synchronization could improve reprogramming efficiency. Utilizing the immunostaining analysis, we calculated the TRA-1–60 and Nanog positive clones on day 14. The results revealed that synchronization (18 h starvation and release for 18 h) dramatically enhanced TRA-1–60 positive clones, which reached about 1.5% (HDF) or 3% (ASC) of parental cells (Figure 3E). In contrast, we observed only 11 (in HDF) and 18 (in ASC) TRA-1–60 positive clones in 10,000 control cells, consistent with previous reports [1], [12], [13]. The expression of Nanog is usually considered an essential pluripotency marker of complete reprogramming. As shown in Figure 3F, Nanog positive clones increased to 1.4% in HDF and 2.6% in ASC after synchronization (15–20 fold, compared with control), which were comparable to previous studies (Table S1). In addition, infected ASC displayed a more homologous morphology than HDF (Figure 3A, B) and achieved a higher reprogramming efficiency accordingly (Figure 3E, F). Our data are in agreement with a recent report that reprogramming human ASC is more efficient than reprogramming adult fibroblasts [21]. These results support the concept that cell cycle synchronization exerts potent effects to drive MET and leads directly to pluripotency.

### 4. Exhibition of hESCs related characters in iPSCs

**Figure 4:**
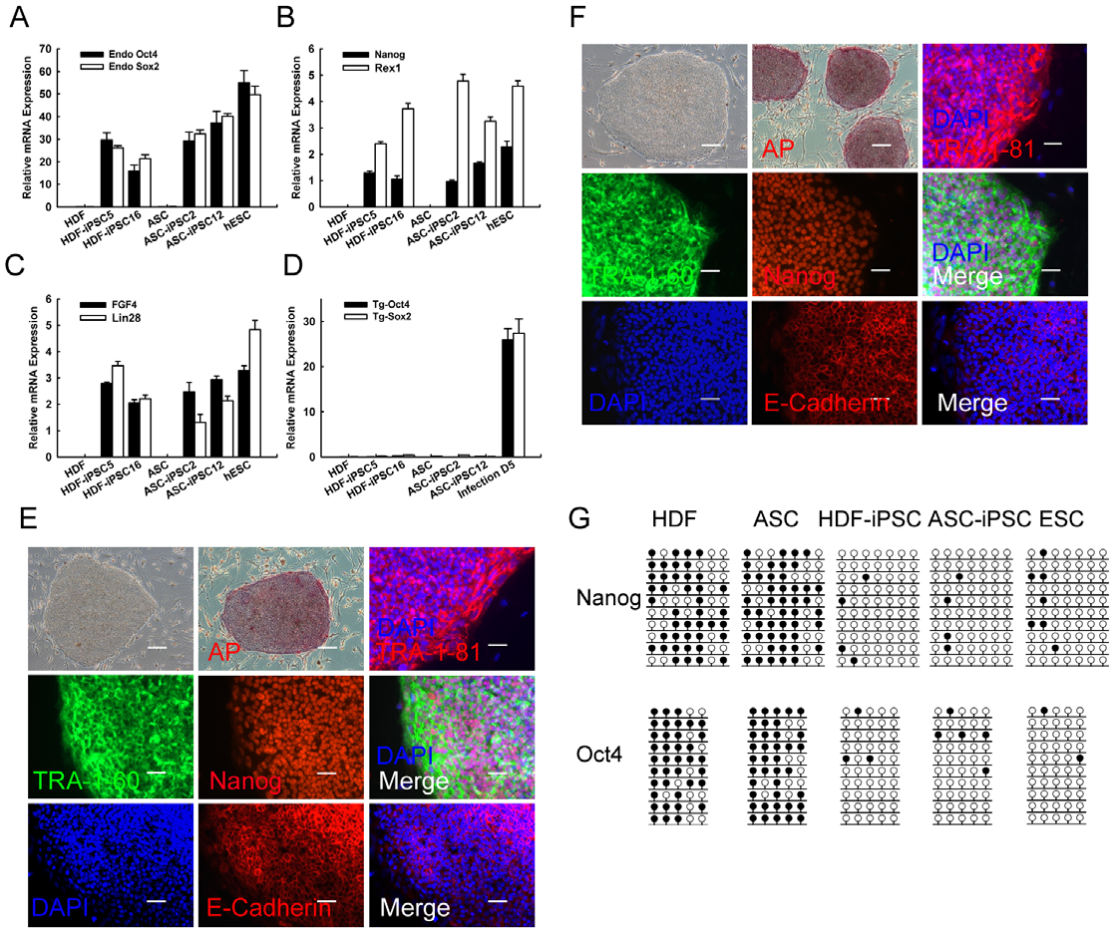
Exhibition of hESCs related characters in hiPSCs. (A): Quantitative PCR assay revealed the relative expression of endogenous Oct4, Sox2 in iPSC lines and ESCs. The data are shown as relative averages ± SEM calculated from triplicate samples. (B): Expression of endogenous Nanog and Rex1 in HDF and ASC derived iPS cell lines. The parental cells of origin and hESCs were used as controls. (C): Quantitative PCR for relative expression of FGF4 and Lin28 in hiPS cell lines. (D): Results from quantitative PCR indicated that exogenous Oct4 and Sox2 transgenes were silenced in hiPSCs. Original cells and cultures on day 5 after retroviral infection were used as controls. (E) and (F): Reprogrammed hiPSCs exhibited hESC morphology and expressed AP (alkaline phosphatase). Scale bar = 200 µm. Immunofluorescence analysis demonstrated that HDF-hiPSCs (E) and ASC-iPSCs (F) expressd TRA-1–81, TRA-1–60, Nanog, and E-Cadeherin. DAPI was used to mark nuclei. Scale Bars = 50 µm. (G): Bisulfite genomic sequencing analysis of Oct4 and Nanog promoters in HDF, ASC, iPSCs, and hESCs.

The mixing of partially reprogrammed clones with genuine iPSCs after infection usually causes much confusion for subsequent iPSCs selection. Previous reports have demonstrated that fully reprogrammed clones exhibit typical characteristics of hESCs, such as a defined boundary and high nucleus-to-cytoplasm ratio within individual cells. Unfortunately, these morphology standards are usually shown to be inefficient. Using a living cell staining strategy to mark TRA-1–60 positive cells, as in a previous publication [21], we generated over 30 human iPSC lines. Most of the iPSC lines were sustained over 35 passages with normal karyotype (Figure S1E). As fully reprogrammed iPSCs are typified by the activation of certain key pluripotency markers and epigenetic reprogramming, we therefore set out to further examine the characteristics of established iPSC lines. Quantitative PCR results verified that all selected iPSC lines activated expression of ESC related pluripotent genes, including Oct4, Sox2, Nanog, Rex1, FGF4, and Lin28 (Figure 4A, B, C). Meanwhile, the silencing of transgenes such as Oct4 and Sox2 was observed (Figure 4D).

Reprogrammed hiPSCs derived from synchronized somatic cells exhibited hESC morphology and expressed AP (Figure 4E, F). Immunofluorescence analysis demonstrated that HDF-hiPSCs (Figure 4E) and ASC-iPSCs (Figure 4F) expressed ESC related markers, including TRA-1–81, TRA-1–60, Nanog, and E-Cadeherin. It has been demonstrated that complete reprogramming is accompanied by extensive epigenetic remodeling, such as DNA demethylation of pluripotency genes in promoter regions [30]. Active demethylation is widely used to monitor successful reprogramming. To further evaluate the epigenetic status of iPSCs, we utilized bisulfite genomic sequencing analysis. Our results revealed that iPSCs derived from synchronized HDF or ASC displayed high demethylation in Oct4 and Nanog promoter regions, which was comparable to ESCs (Figure 4G), whereas the methylation profiles were unmodified in parental cells.

### 5. Differentiation of iPSCs in vitro and in vivo

**Figure 5:**
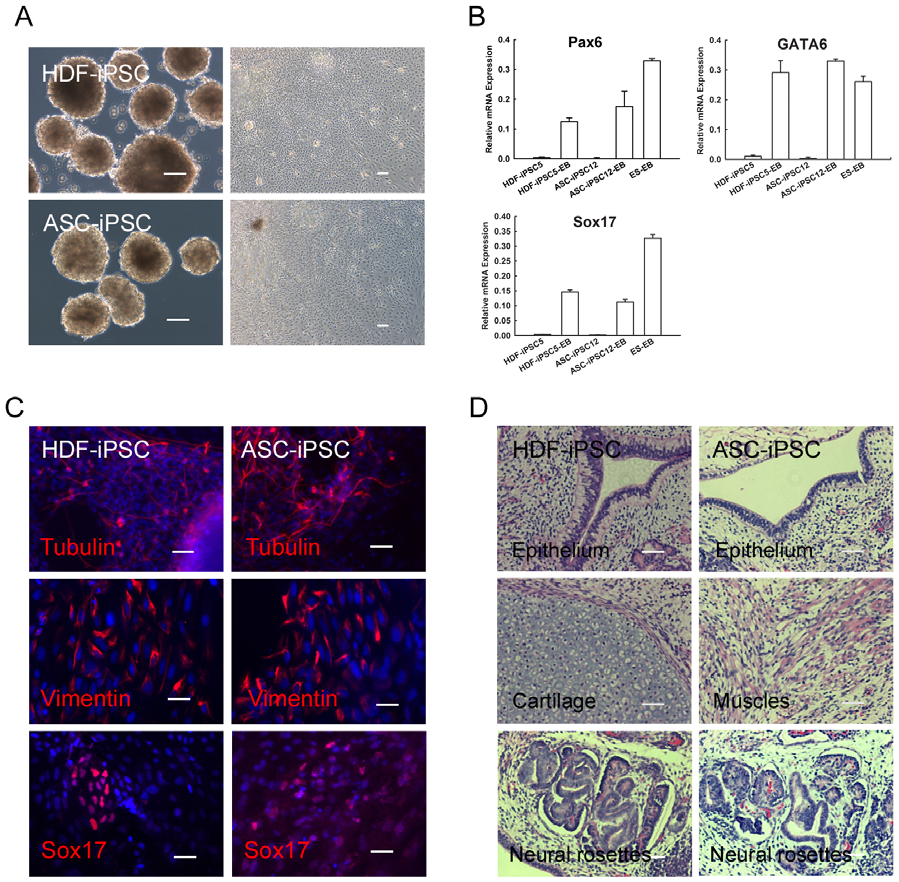
Differentiation of iPSCs in vitro and in vivo. (A): EBs were generated from HDF or ASC derived iPSCs by suspension culture, and auto-differentiation appeared after withdrawing bFGF (right). Scale Bars = 100 µm. (B): Quantitative PCR for specific differentiation markers of the three germ layers. Differentiated iPSCs express Pax6 (ectodermal), GATA6 (mesodermal), and Sox17 (endodermal). (C): Immunostaining for the indicated markers of three germ layers after EB differentiation. Scale Bars = 50 µm. (D): H&E staining showed that the teratomas induced by iPSCs contained tissues of three germ layers. HDF and ASC derived iPSCs differentiation into various tissues including epithelium (endoderm), cartilage (mesoderm), muscles (mesoderm) and neural rosettes (ectoderm). Scale Bars = 50 µm.

iPSCs preserve the ability to differentiate into all cell types of the three germ lines. To evaluate the differentiation potential of iPSCs in vitro and in vivo, synchronized HDF and ASC derived iPSCs were induced to differentiate by embryoid bodies (EBs) or teratoma formation. As shown in Figure 5A, EBs were generated from iPSCs and displayed differentiated morphology after withdrawing bFGF. Quantitative PCR results confirmed that differentiated iPSCs expressed markers of all three germ layers such as Pax6, GATA6, and Sox17 (Figure 5B). We next examined the protein expression by immunofluorescence after EBs differentiation. The results validated that differentiated iPSCs were positive for β-tubulin III (ectoderm), Vimentin (mesoderm), and Sox17 (endoderm) (Figure 5C).

For in vivo differentiation test for pluripotency, both HDF and ASC derived iPSCs were transplanted into SCID mice to induce teratomas. All of the selected iPSC lines (6 HDF-iPSC and 5 ASC-iPSC lines) generated teratomas after injection for 6–8 weeks. H&E staining showed that the teratomas induced by iPSCs differentiated into various tissues including epithelium, cartilage, muscles, and neural rosettes (Figure 5D). These results solidly confirmed that iPSCs derived from synchronized somatic cells were completely reprogrammed into a pluripotent state.

## Discussion

iPS cells provide a unique platform to study the molecular mechanisms of pluripotency and nuclear reprogramming. Compared to mouse cells, however, the process of reprogramming human somatic cells is more time-consuming and less efficient. Even though it is well established that the reprogramming process would involve epigenetic and metabolic remodeling, the molecular regulation underlying the reprogramming still remains largely mysterious [30].

Recently, several small molecules have been reported to enhance reprogramming or substitute for reprogramming factors. These chemicals exert positive effects through epigenetic modifications or signal transduction pathways. Nevertheless, it is necessary to rigorously test the side effects as well. Some modifications generated by chemicals may result in genetic aberrations and gene dysregulation [31].

Cell division is a key parameter for driving epigenetic reprogramming to pluripotency. Inhibition of the P53/P21 pathway or over-expression of Lin28 increases the cell division rate and accelerates the kinetics of iPSCs formation [32]. A recent report has shown that high proliferation rate is an early event in reprogramming, whereas cell cycle arrest by p15, p16, or p21 inhibits retrovirus induced reprogramming [33]. Accelerated cell division could amplify the number of target cells in which each daughter cell has an independent probability of becoming an iPSC, or DNA replication may be the prerequisite for permitting the epigenetic changes, such as histone modifications, to allow the transitions to pluripotency [30]. These studies provided convincing evidence those molecular events in early cell cycle during reprogramming may play a key role in achieving pluripotency.

It is well known that efficient transduction of reprogramming factors is essential for pluripotency. Using an dox-inducible transgenic system, the reprogramming process has been demonstrated to require 10–12 days of sustained transgene expression to activate pluripotency markers [34]. Insufficient infection with partial factors, for example, with only Oct4 and c-Myc, usually results in partial reprogramming [11]. In this study, we demonstrated that synchronization generated a reversible cell cycle arrest at G0/G1 and achieved a coincident cell cycle rhythm, which increased the infection efficiency. Based on this protocol, we significantly improved the reprogramming efficiency to 1.4% in HDF and 2.6% in ASC (Nanog positive clones, Figure 3F, Table S1). This synchronization strategy can also be used to promote retrovirus mediated transgene expression in other research contexts.

During the process of reprogramming, the target cells undergo a dramatic morphological change from a mesenchymal phenotype to an ES-like epithelial feature [35]. MET is also considered a hallmark of the initial phase and necessary for reprogramming [29], [36]. It is noted that BMP signaling synergizes with OSKM to promote MET [37]. Interestingly, our results showed that synchronized HDF and ASC displayed uniform MET after infection and facilitated human somatic reprogramming. A possible interpretation is that synchronized cells (prior to G2/M phase) tend to be infected by retroviruses, which leads to more efficient expression of transgenes. In addition, synchronization creates the possibility of simultaneous infection in most cells, followed by a synchronized expression of reprogramming factors in individual cells. Finally, certain auto- or intercellular regulation after synchronized expression of transgenes may trigger a homologous MET and direct to pluripotency.

It is difficult to distinguish full and partial reprogramming by means of cell morphology, because the partial clones are far more numerous than the ES-like ones. It has been reported that reprogramming is a stochastic process, and most infected cells were trapped in a partially reprogrammed state due to inability to overcome the epigenetic barrier [4]. Thus, a detailed study of transcriptional and epigenetic resetting is essential to exclude partial reprogramming. In somatic cells, the promoters of Oct4 and Nanog are highly methylated, reflecting repression at the transcriptional level. Our results revealed that synchronized HDF and ASC derived iPSCs showed high demethylation in Oct4 and Nanog promoter regions.

Since human iPSC are similar to ESC, we chose ESC derived EB as a control to test the differentiation ability of iPSC, as previously described [7], [38]. We did find that the expression of Pax6 and Sox17 (except in GATA6) was lower than ES-EB, even though all of the selected iPSC lines in our study can generate teratoma, a gold standard of pluripotency [39]. These results are in agreement with previous conclusion that human iPSC is similar but not identical to ESC [1]. It is also acceptable that iPSC tend to redifferentiate into the derived donor cells [40], [41], as shown in our results (Figure 5 B) a relative higher GATA6 expression was detected. Previous results have also shown the unequal expression levels in different iPSC lines after differentiation [7], [38], [42].

In conclusion, our results provide direct evidence that cell cycle synchronization greatly promotes MET and reprogramming efficiency without the need for any additional chemicals. Our findings offer new insights into the process of reprogramming and will be beneficial to the efficient generation of disease-specific iPSCs.

## Materials and Methods

### Ethics Statement

Human tissues used in this study were obtained from healthy individuals after circumcision or lipoaspiration. All human samples were collected with patients' informed consent and the experimental protocols were approved (Approval number: 2010022601H) by the institutional review board of Zhongshan Ophthalmic Center, Sun Yat-sen University.

### Cell Culture

Human embryonic stem cells (hESCs) H9 were obtained from Wicell research institute. hESCs and hiPSCs were maintained on mitomycin C-inactived mouse embryonic fibroblasts (MEF, Millipore) in hES medium: Knockout DMEM (Invitrogen) supplemented with 20% knockout serum replacement (Invitrogen), 10 ng/ml bFGF (PeproTech), 1 mM L-Glutamine, 1×10^–4^ M nonessential amino acids, 0.1 mM beta-mercaptoethanol, 50 units/ml penicillin, and 50 mg/ml streptomycin (Invitrogen). Primary HDF and ASC were cultured in DMEM/F12 (Gibco) supplemented with 20% FBS (Hyclone), 1 mM L-Glutamine, 1×10^–4^ M nonessential amino acids, 0.1 mM beta-mercaptoethanol, 50 units/ml penicillin, and 50 mg/ml streptomycin (Invitrogen). More information about isolation of HDF and ASC was provided in Methods S1.

### Synchronization procedure and cell cycle analysis

To synchronize the cell cultures, HDF and ASC were seeded in 60 mm dishes in growth medium with 20% FBS overnight. Then the cultures were rinsed by PBS and changed to serum free medium. After serum starvation for 12, 18, or 24 h, the cells were passaged and released into cell cycle by addition of serum [24]. For FACS analysis, cell samples were harvested with trypsinization at indicated time points. The staining procedures were performed using a BD Cycletest Plus DNA Reagent Kit according to manufacture's instructions. Cell cycle phase distributions were analyzed by flow cytometry (BD Bioscience). In addition, the percentage of cells in each phase of cell cycle was analyzed by Modfit software (Verity).

### BrdU incorporation assay and apoptosis assay

5-bromo-2′deoxyuridine (BrdU) incorporation assay was conducted to monitor DNA replication using Cell Proliferation Fluorescence Kit (GE). As manufacture's instructions, HDF or ASC were seeded on cover slips, after allowing to incorporating BrdU for 1 h, the samples were fixed with 4% paraformaldehyde/0.1% Triton X-100/PBS for 20 min, and then incubated with anti-BrdU/Nuclease and Alexa Fluor 488 labeled anti mouse IgG sequentially.

To examine the side effects of serum starvation on cell viability, Hoechst 33258 (Invitrogen) staining was performed to determine the proportion of apoptotic cells at the indicated time. Confocal images were acquired with a laser scanning microscope (LSM 510, Zeiss), and a minimum of 500 cells were scored to calculate apoptosis rate. Morphological changes including chromatin condensation and nuclei fragmentation were considered as apoptotic cell according to a previous report [43].

### Retroviral infection and induced reprogramming

The detailed experiment procedure for retrovirus packaging is provided in the Methods S1. The day before infection, cell cycle synchronized HDF or ASC were seed into 12-well plate at 3×10^4^ per well. For infection, 0.6–0.8 ml concentrated retrovirus (mixture of 4 factors), 0.5 ml growth medium, and 4 µg/ml polybrene (sigma) were added into cells. After incubation for 24 h, the medium was replaced with 1 ml fresh growth medium. 3–5 days after infection, the morphologically epithelial-like cells appeared. Infected cells were digested, 3–4×10^4^ cells were transferred to 100 mm dish with MEF on day 5–6. The medium were replaced with hES medium from day 9, the hES-like colonies emerged from day 12–14.

To pick up pluripotent clones, the cultures were marked by live cell staining from day 12 to 16 as previously described [21]. Briefly, the cells were washed with prewarmed PBS, and then incubated directly with TRA-1–60 (1∶50 dilution, Millipore) and appropriate secondary antibody in phenol red free DMEM (Invitrogen). Washing cells 2 times and live images were obtained in the phenol red free media. The TRA-1–60 positive hES-like colonies were marked under a fluorescence microscope and transferred to 12-well plates with feeder cells.

### Immunofluorescence

For immunofluorescence analysis, cell samples were fixed in 4% paraformaldehyde for 10–15 min and rinsed with PBS, then permeabilized and blocked in 0.1% Triton X-100/PBS containing 3% BSA for 30 min. After incubation with primary antibodies for 2 h at room temperature or overnight at 4°C, the samples were washed three times in PBS, and subsequently incubated with Cy3 or Alexa Fluor 488 labeled secondary antibody for 1 h. Negative controls were stained without primary antibodies. After washing three times with PBS, samples were counterstained with DAPI (Invitrogen).

The following antibodies were used: rabbit anti-Nanog (1∶200 dilution, Abcam), mouse anti-Oct3/4 (1∶100 dilution, Millipore), mouse anti-Sox2 (1∶100 dilution, Millipore), mouse anti-TRA-1–60 (1∶100 dilution, Millipore), mouse anti-TRA-1–81 (1∶100 dilution, Millipore), Rabbit anti-E-cadherin (1∶100 dilution, Cell Signaling), mouse anti-Tuj1 (1∶100 dilution, Millipore), goat anti-Sox17 (1∶100 dilution, R&D), mouse anti-Vimentin (1∶100 dilution, Santa Cruz).

### RT-PCR

Total RNA was extracted using TRI Reagent (Ambion), and then treated with DNase I (sigma) to remove genomic DNA contamination. RevertAid First Strand cDNA Synthesis Kit (Fermentas) was used to synthesize complementary DNA from 2 µg total RNA. For quantitative analysis, 20–50 ng cDNA samples were amplified on LightCycler480 (Roche Diagnostics) by SYBR PrimeScript™ RT-PCR Kit (TaKaRa). Each PCR analysis was performed in 35 cycles, the PCR primer sequences are shown in Table S2. The mRNA expression level of each gene relative to GAPDH (housekeeping gene) was calculated as previously described [44].

### Bisulfite sequencing analysis

Genomic DNA was extracted to analyze the methylation pattern in iPSC lines. Bisulfite treatment was performed using the CpGenome modification Kit (Millipore) according to the manufacture's recommendation. The promoter regions of Oct4 and Nanog were amplified by PCR and subcloned into pGEM-T (Promega). Ten clones of each sample were verified by sequence analysis. The methylation status was analyzed by CpGViewer software. The PCR primer sequences are shown in Table S2.

### In vitro differentiation and Teratoma formation

For in vitro differentiation, HDF and ASC derived iPSCs were floating cultured in hESC medium without bFGF for EBs formation. After 4–6 days, EBs were transferred to 0.1% gelatin-coated 6-well plates in DMEM/F12 containing 20% FBS for spontaneous differentiation. To induce neuron differentiation, EBs were cultured in medium containing N2 and B27 supplement (Invitrogen). The differentiated cultures were stained with appropriate antibody and followed by immunofluorescence analysis.

For teratoma formation, HDF and ASC derived iPSCs were collected by collagenase IV digestion, and resuspended at 5×10^6^ cells/ml in 200 µl matrigel (BD Biosciences) and 20% FBS. The cell suspension was subcutaneously transplanted into SCID mice (Laboratory Animal center of Sun Yat-sen University). Tumors were appeared from 6–8 weeks after injection. For histological analysis, mice were euthanized at 9–10 weeks, and teratomas were dissected from the host. After fixed in 4% paraformaldehyde, the sections were processed with hematoxylin and eosin (HE) staining. The animal experiments were performed and approved (Approval number: 2009112303) in accordance with Zhongshan Ophthalmic Center Guidelines of Animal Care and Use Committee.

### Statistical analysis

Data were represented as mean ± SEM from 3 independent experiments (see figure legends for specific details). Statistical analysis of the data was performed using SPSS13.0, and significance of differences was examined with Student *t* test. *P*<0.05 was considered statistically significant.

## Supporting Information

Figure S1 **hiPS cells sustained a normal karyotype.** (A): FACS data showed that ASC were rendered G0/G1 phase by serum deprivation. *P<0.01 *vs* FBS control (n = 3). (B): Synchronization in ASC increased BrdU positive cells. (C): Morphology of HDF after serum deprivation. (D): Confocal images showed Hoechst 33258 in control and 24 h starved HDF exhibited homogeneous nuclear staining. Apoptotic cells with condensed chromatin (arrowhead) or fragmented nuclei (arrow) were detected after starvation for 48 h. (E): Representative images showing iPS cell lines derived from HDF (left) and ASC (right) sustained normal 46 XY karyotype.
(TIF)

Table S1 **Summary of reprogramming efficiency in human adult cells.**
(DOC)

Table S2 **Primers for RT-PCR and bisulfite sequencing.**
(DOC)

Methods S1 **Supporting methods for cell culture, retroviral packaging and karyotype analysis.**
(DOC)
