# Supplementary figures and images for: Serum Starvation Induced Cell Cycle Synchronization Facilitates Human Somatic Cells Reprogramming
Source: PLoS One. 2012 Apr 18;7(4):e28203. doi: 10.1371/journal.pone.0028203 (PMC3329488; doi:10.1371/journal.pone.0028203)

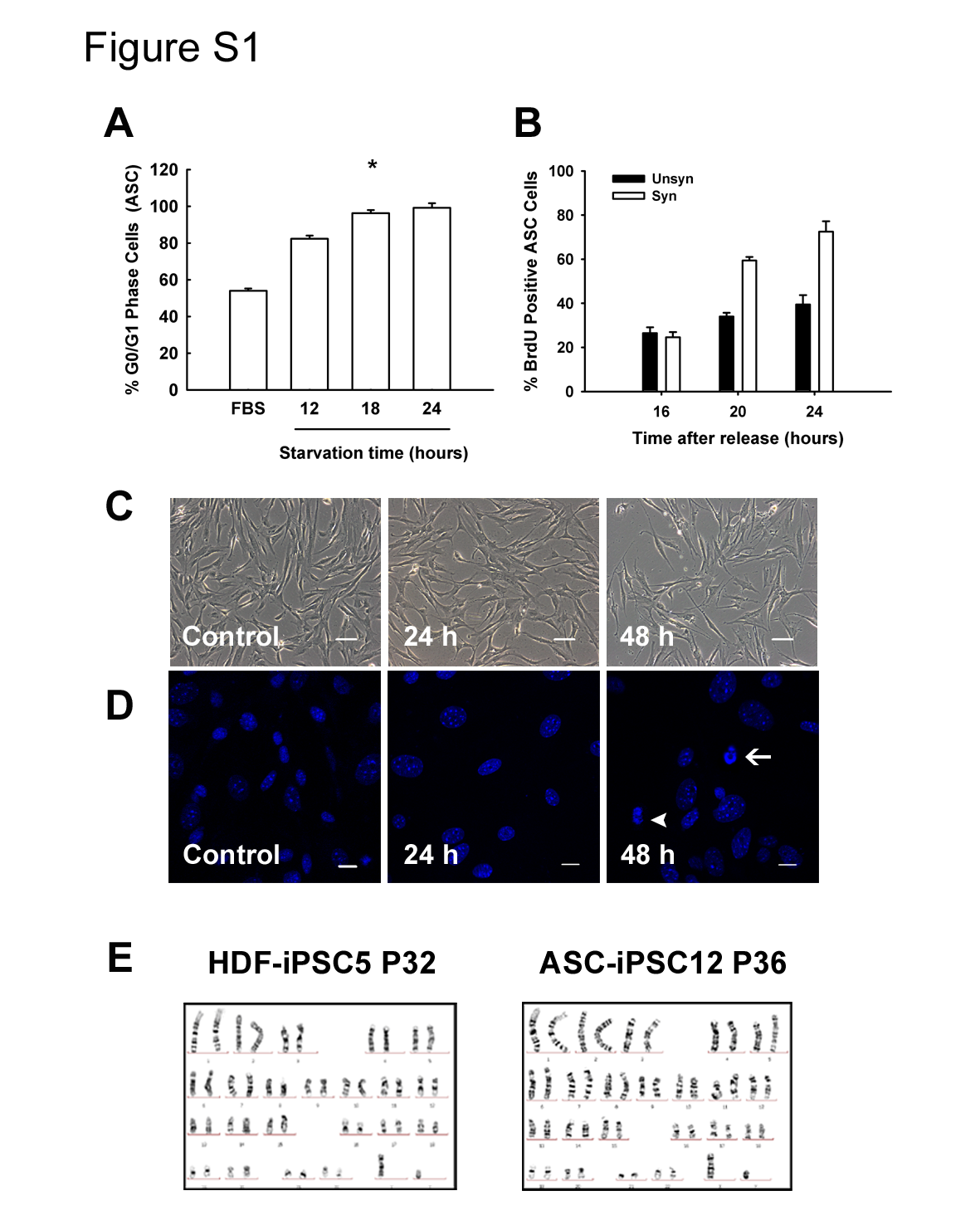

Supplement: Figure S1 — hiPS cells sustained a normal karyotype. (A): FACS data showed that ASC were rendered G0/G1 phase by serum deprivation. *P<0.01 vs FBS control (n = 3). (B): Synchronization in ASC increased BrdU positive cells. (C): Morphology of HDF after serum deprivation. (D): Confocal images showed Hoechst 33258 in control and 24 h starved HDF exhibited homogeneous nuclear staining. Apoptotic cells with condensed chromatin (arrowhead) or fragmented nuclei (arrow) were detected after starvation for 48 h. (E): Representative images showing iPS cell lines derived from HDF (left) and ASC (right) sustained normal 46 XY karyotype. (TIF) [file pone.0028203.s001.tif]
