# Supplementary material for: Serum Starvation Induced Cell Cycle Synchronization Facilitates Human Somatic Cells Reprogramming
Source: PLoS One. 2012 Apr 18;7(4):e28203. doi: 10.1371/journal.pone.0028203 (PMC3329488; doi:10.1371/journal.pone.0028203)
Supplement: Table S1 — Summary of reprogramming efficiency in human adult cells. (DOC) [file pone.0028203.s002.doc]

**Table S1. The efficiency of reprogramming human adult cells in previous studies**

| Reprogramming factors | Chemical additive | Cell type | Scoring criteria | Reprogramming efficiency | reference |
| --- | --- | --- | --- | --- | --- |
| Retroviral vector  OCT4, SOX2, KLF4, c-MYC | None | Human dermal fibroblasts | ESC-like clones | 0.02% | (Takahashi et al. 2007) |
| Retroviral vector  OCT4, SOX2, KLF4, c-MYC | Y27632 | ES-cell-derived fibroblasts | ESC-like clones | 0.1% | (Park et al. 2008) |
| Retroviral vector  OCT4, SOX2, KLF4 | VPA | Human neonatal fibroblasts | ESC-like clones | 1.1% | (Huangfu et al. 2008) |
| Retroviral vector  OCT4, SOX2, KLF4, c-MYC | None | Human keratinocytes | AP positive hESC like clones | 0.76% | (Aasen et al. 2008) |
| Retroviral vector  OCT4, SOX2, KLF4, c-MYC | Vitamin C  and VPA | Human dermal fibroblasts | AP positive hESC like clones | 6.2% | (Esteban et al. 2010) |
| Retroviral vector  OCT4, SOX2, KLF4, c-MYC | Vitamin C  and VPA | Human adipose stem cells | AP positive hESC like clones | 7.06% | (Esteban et al. 2010) |
| Retroviral vector  OCT4, SOX2, KLF4, c-MYC | Butyrate | IMR90 fibroblasts | TRA-1-60 positive clones | 15-20% | (Mali et al. 2010) |
| Retroviral vector  OCT4, SOX2, KLF4, c-MYC | None | Human astrocytes | Nanog positive clones | 0.1% | (Ruiz et al. 2010) |
| Retroviral vector  OCT4, SOX2, KLF4, c-MYC | SB431532 | human umbilical vein endothelial cells | Nanog positive clones | 2.5–3% | (Panopoulos et al. 2011) |
| Lentiviral vector  OCT4, SOX2, KLF4, c-MYC | None | Human adipose stem cells | TRA-1-60 positive clones | 0.2% | (Sun et al. 2009) |
| Retrovirus  OCT4, SOX2, KLF4, c-MYC | None | Human dermal fibroblasts | Nanog positive clones | 1.4% | Present study |
| Retrovirus  OCT4, SOX2, KLF4, c-MYC | None | Human adipose stem cells | Nanog positive clones | 2.6% | Present study |
