# Supplementary material for: Serum Starvation Induced Cell Cycle Synchronization Facilitates Human Somatic Cells Reprogramming
Source: PLoS One. 2012 Apr 18;7(4):e28203. doi: 10.1371/journal.pone.0028203 (PMC3329488; doi:10.1371/journal.pone.0028203)
Supplement: Table S2 — Primers for RT-PCR and bisulfite sequencing. (DOC) [file pone.0028203.s003.doc]

**Table S2. Primer sequences used in RT-PCR**

| **Gene name** | **Primer sequences (5'-3')** | **Application** |
| --- | --- | --- |
| hOct3/4-F  hOct3/4-R | GACAGGGGGAGGGGAGGAGCTAGG  CTTCCCTCCAACCAGTTGCCCCAAAC | Endo RT-PCR |
| hSox2-F  hSox2-R | GGGAAATGGGAGGGGTGCAAAAGAGG  TTGCGTGAGTGTGGATGGGATTGGTG | Endo RT-PCR |
| hNanog-F  hNanog-R | CCAACATCCTGAACCTCAGC  GCTATTCTTCGGCCAGTTG | hNanog RT-PCR |
| hFGF4-F  hFGF4-R | CTACAACGCCTACGAGTCCTACA  GTTGCACCAGAAAAGTCAGAGTTG | hFGF4 RT-PCR |
| hLin28-F  hLin28-R | GAAGCGCAGATCAAAAGGAG  GCTGATGCTCTGGCAGAAGT | hLin28 RT-PCR |
| hRex1-F  hRex1-R | CAGATCCTAAACAGCTCGCAGAAT  GCGTACGCAAATTAAAGTCCAGA | hRex1 RT-PCR |
| hCdh1-F  hCdh1-R | TGCCGCCATCGCTTACAC  CGACGTTAGCCTCGTTCTCAG | hCdh1 RT-PCR |
| hEpcam-F  hEpcam-R | GGCTTTATGATCCTGACTGCG  TCCTTGTCTGTTCTTCTGACCC | hEpcam RT-PCR |
| hSnail-F  hSnail-R | CCAATCGGAAGCCTAACTACAGCG  AAGGACGAAGGAGCCGGTGAG | hSnail RT-PCR |
| hCdh2-F  hCdh2-R | GGCGGAGATCCTACTGGACGG  TGACTGAGGCGGGTGCTGAAT | hCdh2 RT-PCR |
| hPax6-F  hPax6-R | ACCCATTATCCAGATGTGTTTGCCCGAG  ATGGTGAAGCTGGGCATAGGCGGCAG | hPax6 RT-PCR |
| hSox17-F  hSox17-R | CGCTTTCATGGTGTGGGCTAAGGACG  TAGTTGGGGTGGTCCTGCATGTGCTG | hSox17 RT-PCR |
| hGATA6-F hGATA6-R | GCGGGCTCTACAGCAAGATG  ACAGTTGGCACAGGACAATCC | hGATA6 RT-PCR |
| GAPDH-F  GAPDH-R | TGTGATGGGTGTGAACCACGAG  TGGGAGTTGCTGTTGAAGTCGC | hGAPDH RT-PCR |
