# Supplementary material for: Serum Starvation Induced Cell Cycle Synchronization Facilitates Human Somatic Cells Reprogramming
Source: PLoS One. 2012 Apr 18;7(4):e28203. doi: 10.1371/journal.pone.0028203 (PMC3329488; doi:10.1371/journal.pone.0028203)
Supplement: Methods S1 — Supporting methods for cell culture, retroviral packaging and karyotype analysis. (DOC) [file pone.0028203.s004.doc]

**Serum starvation induced cell cycle synchronization facilitates human somatic cells reprogramming**

**Supporting Methods** **S1**

**Cell culture**

Primary HDF and ASC were cultured as previously described [1, 2]. For HDF separation, foreskin tissues were washed in cold phosphate-buffered saline (PBS) and dissected into 1 mm size pieces, then plated within 100 mm dishes contain 3 ml FBS. After incubation at 37 ºC, 5% CO_2_ overnight, 5 ml growth medium were added. Outgrowth of fibroblasts was appeared after 5-8 d. For ASC isolation, adipose tissues were harvested from lipoaspiration and digested in collagenase type II for 30 min. ASC were collected by filtering through 100 μm cell strainer (BD Bioscience). HDF and ASC were following expanded in DMEM/F12 (Gibco) supplemented with 20% FBS (Hyclone), 1 mM L-Glutamine, 1×10^–4^ M nonessential amino acids, 0.1 mM beta-mercaptoethanol, 50 units/ml penicillin, and 50 mg/ml streptomycin (Invitrogen).

To measure population doubling time, cells (Passage 1) were seeded in triplicate at a density of 5×10^3^/cm^2^, cell number was determined every 48 h after trypsinization. Population doubling times were calculated as previous report [3].

**Retroviral packaging**

Retrovirus packaging and infection were carried out according to previous publication [4]. Briefly, 293T cells (ATCC) were seeded at 3.6×10^6^ per 100 mm dishes 1 day before transfection. Reprogramming factors pMXs-OCT3/4, pMXs- SOX2, pMXs-cMYC, and pMXs-KLF4 (Addgene) were transfected with retrovirus packaging vectors VSV-G and GP. For each transfection, the DNA-Fugene HD mixture containing 400 μl opti-MEM (Invitrogen), 12 μg reprogramming factor, 7.5 μg GP, 4.5 μg VSV-G, and 54 μl Fugene HD (Roche). The medium was replaced with 15ml fresh medium after overnight incubation. Virus-containing supernatants were collected at 48 h, 72 h, and 96 h after transfection, and then mixed for filtered through a 0.45 μm cellulose acetate filter (Sartorius). To obtain higher-titer retroviruses, the supernatants were subsequently concentrated with Amicon Ultra-15 Centrifugal Filter Units (Millipore). Using this protocol, we obtained about 10-fold concentrated viruses. A pMXs-GFP retrovirus was generated in parallel as infection control.

**Karyotype analysis**

For karyotype analysis, HDF and ASC derived iPSCs were maintained on matrigel without feeder cells. Cells were treated with 0.2 μg/ml of colcemid for 3 hours and subsequently harvested by trypsin. After treatment with hypotonic solution and fixation with 3:1 methanol and acetic acid, the samples were analyzed by the standard G-banding method.

**References**

1. Sun N, Panetta NJ, Gupta DM, Wilson KD, Lee A, et al. (2009) Feeder-free derivation of induced pluripotent stem cells from adult human adipose stem cells*.* Proc Natl Acad Sci U S A 106: 15720-15725.

2. Park IH, Lerou PH, Zhao R, Huo H, and Daley GQ (2008) Generation of human-induced pluripotent stem cells*.* Nat Protoc 3: 1180-1186.

3. Mohamet L, Lea ML, and Ward CM (2010) Abrogation of E-cadherin-mediated cellular aggregation allows proliferation of pluripotent mouse embryonic stem cells in shake flask bioreactors*.* PLoS One 5: e12921.

4. Mali P, Chou BK, Yen J, Ye Z, Zou J, et al. (2010) Butyrate greatly enhances derivation of human induced pluripotent stem cells by promoting epigenetic remodeling and the expression of pluripotency-associated genes*.* Stem Cells 28: 713-720.
